# Flight Test Result for the Ground-Based Radio Navigation System Sensor with an Unmanned Air Vehicle

**DOI:** 10.3390/s151128472

**Published:** 2015-11-11

**Authors:** Jaegyu Jang, Woo-Guen Ahn, Seungwoo Seo, Jang Yong Lee, Jun-Pyo Park

**Affiliations:** The 3rd R&D Institute-4, Agency for Defense Development, Yuseong P.O. Box 35, Daejeon 305-600, Korea; E-Mails: wgahn@add.re.kr (W.-G.A.); mcnara82@add.re.kr (S.S.); flukelee@add.re.kr (J.Y.L.); pjp1023@add.re.kr (J.-P.P.)

**Keywords:** pseudolite, pulsed signal, false lock

## Abstract

The Ground-based Radio Navigation System (GRNS) is an alternative/backup navigation system based on time synchronized pseudolites. It has been studied for some years due to the potential vulnerability issue of satellite navigation systems (e.g., GPS or Galileo). In the framework of our study, a periodic pulsed sequence was used instead of the randomized pulse sequence recommended as the RTCM (radio technical commission for maritime services) SC (special committee)-104 pseudolite signal, as a randomized pulse sequence with a long dwell time is not suitable for applications requiring high dynamics. This paper introduces a mathematical model of the post-correlation output in a navigation sensor, showing that the aliasing caused by the additional frequency term of a periodic pulsed signal leads to a false lock (*i.e*., Doppler frequency bias) during the signal acquisition process or in the carrier tracking loop of the navigation sensor. We suggest algorithms to resolve the frequency false lock issue in this paper, relying on the use of a multi-correlator. A flight test with an unmanned helicopter was conducted to verify the implemented navigation sensor. The results of this analysis show that there were no false locks during the flight test and that outliers stem from bad dilution of precision (DOP) or fluctuations in the received signal quality.

## 1. Introduction

GNSS (Global Navigation Satellite System) receivers are currently the most widely-used space-based PNT (positioning, navigation and timing) sensors. From infrastructure, such as the time-keeping systems of wireless telecommunication devices, to mobile devices or military weapons, they are now indispensable. However, the vulnerability of the satellite navigation systems has been an important issue for more than a decade, as GPS sensors must process signals with an extremely low power level. The seriousness of this vulnerability can be found in a statement issued by the Interagency GPS Executive Board, which reads “GPS users must ensure that adequate independent backup systems or procedures can be used when needed” [1]. For the past few years, there have in fact been attacks on the GPS L1-band close to the boarder of Korea, with one article stating that “aircraft had to rely on alternative navigation aids” during the attacks [2].

The ground-based radio navigation system introduced in this paper is an alternative/backup navigation system, which can overcome the vulnerability against intentional interference attacks through its use of a pseudolite network. Before the launching of GPS (Global Positioning System) satellites, pseudolites were used to test transmitters at the desert test ranges [3]. For the last twenty years, many researchers have extended the applications of these systems to various areas, including indoor positioning, the Mars navigation system and regional positioning systems [4,5,6,7,8,9]. The ground-based radio navigation system has been studied as a regional positioning system of which the main applications are air vehicles. This type of system belongs to the synchronized pseudolite navigation system category, and these systems have a better system survivability than those in the asynchronized system category [4,10].

In a CDMA (code division multiple access)-based system, users share the same frequency channel. This means that users can fail to acquire weak signals if there are strong signals from closely-located transmitters. This is known as the near-far issue. To avoid the near-far problem in the pseudolite sensors, previous researchers suggested a scheme that involved frequency offsetting and pulsing [11,12]. We applied these two methods to avoid the near-far issue in our study. By applying the pulsing, we can allocate time slots to each signal, which means that there are no more collisions between signals. Earlier suggestions, such as RTCM SC-104 and RTCA (radio technical commission for aeronautics) 2000, recommended a randomized sequence, because the pulsed signal may have an aliasing effect on the navigation sensor. Because an additional frequency term caused by a periodic pulse could lead to a possible false lock in the tracking loop, they recommended accumulation of the received signal samples with a randomized pulse position. If the accumulation-and-dump time is long enough to use all of the sequence chips, received signal samples will appear to be continuous in the accumulation-and-dump filter. However, this method requires a long dwell time (e.g., at least 10 ms [11]), and the time is typically too long for some applications with high dynamics.

In this study, we designed pseudolite transmitters with fixed pulse positions, for which the duty ratio is defined as 10%. We also suggested another algorithm that detects possible false locks in the navigation sensor. First, we introduce a mathematical model of the accumulation-and-dump filter output for a periodic pulsed signal, after which we explain why a false lock can occur. Secondly, two algorithms are suggested to detect a false lock in the frequency domain. After detecting a false lock with the multi-correlator characteristics, the algorithm performs a Doppler correction in a signal processing module. The implemented firmware was verified in the flight test with an unmanned helicopter. An analysis of the flight test confirmed that the navigation solutions were reliable and that there were no issues caused by the false lock, although there were a few outliers in the solution caused by bad dilution of precision (DOP) at a low elevation and by channel power fluctuations.

## 2. Mathematical Model

According to earlier work [13], the sum of the sine and cosine samples of an incoming continuous signal can be approximated by Equation (1) in a complex form.

(1) $${\langle IQ\rangle }_{\cos/\sin}\approx \int_{0}^{T}e^{j(2\pi \cdot \Delta f\cdot t+\Delta \phi )}dt=\frac{\sin(\pi \cdot \Delta f\cdot T)}{\pi \cdot \Delta f\cdot T}e^{j\Delta \phi }\;$$

Here, *T* is the coherent integration time of the accumulation-and-dump filter, Δ*Φ* is the phase error in radian units and Δ*f* is the frequency error in Hz units.

If the coherent integration time is limited to a code sequence period (or a single pulse period), a redefinition of Equation (1) for aperiodic pulsed navigation signals is straightforward, because the integration time is only affected by the duty ratio of the pulse. Therefore, the accumulation-and-dump output model of the incoming signal can be rewritten as Equation (2).

(2) $${\langle IQ\rangle }_{aperiodic}=\frac{\sin(\pi \cdot \Delta f\cdot DR\cdot T_{seq})}{\pi \cdot \Delta f\cdot DR\cdot T_{seq}}\cdot \sqrt{2\frac{C}{N_{0}}\cdot DR\cdot T_{seq}}\cdot R(\tau )\cdot D\cdot e^{j\Delta \phi }\text{+}\eta \;$$

Here, *T_seq_* is the coherent integration time for a code sequence period, *DR* is the duty ratio of the pulse, *C/N_0_* is the carrier to noise density ratio, *R(τ)* is an auto-correlation function with a code phase error of *τ*, *D* is the data bit sign and *η* denotes the noise in a complex form.

**Figure 1:**
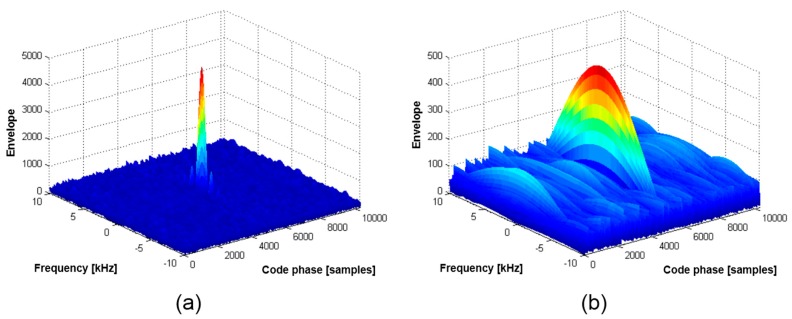
Null-to-null space extension due to a single pulse.

The first term in Equation (2) explains why the null-to-null space of the signal acquisition result in the frequency domain changes when a pulse is applied to an incoming code sequence, as presented in Figure 1. Figure 1a shows the 2D correlation outputs when a coherent integration time of 1 ms is used, as in a GPS navigation signal acquisition, while Figure 1b presents the pulsed signal acquisition result, for which the duty ratio is set to 0.1. Figure 1 shows that the null-to-null frequency space is increased in a manner inversely proportional to the duty ratio *DR*, which is identical to the first term of Equation (2).

**Figure 2:**
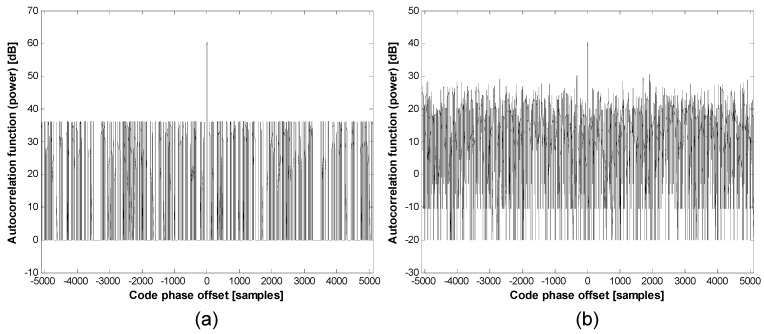
Partial correlation effect on the power: (**a**) duty ratio of 1.0; (**b**) duty ratio of 0.1.

The second term of Equation (2) implies partial correlation effects on the power due to the pulsing scheme acting on a code sequence. Figure 2 shows that the post-correlation power is reduced by a pulse with a duty ratio of 0.1; the second term of Equation (2) explains why in a mathematical expression. Reduced power itself is not critical to a ground-based local navigation system, because it can be resolved by a systematic design, such as transmission power control. The important issue in Figure 2 is the change of the cross-correlation property. As shown in Figure 2, the cross-correlation separation is reduced from 24 to 25 dB to approximately 10 dB by pulsing.

**Figure 3:**
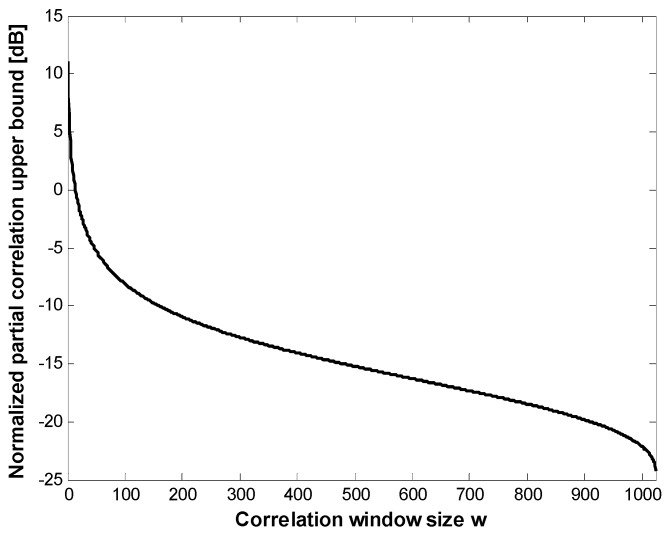
Cross-correlation separation of the pulsed C/A code.

Assuming a Gaussian approximation, the upper bound of the partial correlation (*i.e*., the cross-correlation separation bound) of pulsed C/A Gold codes can be estimated, as shown in Figure 3. If the window size is set to 102–103 chips (*DR* = 0.1), the upper bound approaches −10 dB or more, as simulated in Figure 2.

A new spreading code design to obtain good correlation properties is out of the scope of this paper. Therefore, a coherent integration time over a single code sequence period is unavoidable to meet the sensitivity requirements of navigation sensors in general. In this case, we must utilize the periodic pulse train model. Here, we define a rectangular pulse train signal model with Equations (3) and (4):

(3) $$p_{0}(t)=\{\begin{array}{c} 1,\begin{array}{ccc} & & \left|t\right|\leq DR\cdot T_{seq}/2 \end{array}\\ 0,\begin{array}{ccc} & & DR\cdot T_{seq}/2<\left|t\right|\leq T_{seq}/2 \end{array} \end{array}\;$$

(4) $$p(t)=p_{0}(t\pm n\cdot T_{seq}),\begin{array}{cc} & n=0,\pm 1,\pm 2,... \end{array}\;$$

Equations (3) and (4) correspond to the aperiodic and the periodic model, respectively. The series coefficients of Equation (3) are derived as follows:

(5) $$c_{n}\approx \frac{1}{T_{seq}}\int_{-T_{seq}/2}^{T_{seq}/2}p(t)\cdot e^{-jn\frac{2\pi }{T_{seq}}t}dt=\frac{1}{n\pi }\sin(DR\cdot n\cdot \pi )=\frac{\sin(DR\cdot n\cdot \pi )}{DR\cdot n\pi }\;$$

By inserting Equation (5) into the exponential Fourier series definition, the pulse train in Equation (4) can be rewritten as Equation (6).

(6) $$p(t)=DR\cdot \sum_{n=-\infty }^{\infty }\sin c(n\cdot DR)\cdot e^{jn\frac{2\pi }{T_{seq}}t}\;$$

Equation (1) can be rewritten as Equation (7) by applying Equation (6), as follows:

(7) $$\begin{array}{ll} {\langle IQ\rangle }_{\cos/\sin}^{periodic} & =\int_{0}^{KT_{seq}}e^{j(2\pi \cdot \Delta f\cdot t+\Delta \phi )}\cdot DR\cdot \sum_{n=-\infty }^{\infty }\sin c(n\cdot DR)\cdot e^{jn\frac{2\pi }{T_{seq}}t}dt\\ & =DR\cdot \left[\sum_{n=-\infty }^{\infty }\sin c(n\cdot DR)\int_{0}^{KT_{seq}}e^{j\left\{2\pi (\Delta f+nf_{seq})t+\Delta \phi \right\}}\cdot dt\right] \end{array}$$

Here, *f_seq_* denotes the code sequence frequency in Hz (=1/*T_seq_*). The integral term in Equation (7) resembles the form of Equation (1) exactly; thus, we can easily rearrange Equation (7) above into Equation (8):

(8) $${\langle IQ\rangle }_{\cos/\sin}^{periodic}=DR\cdot \left[\sum_{n=-\infty }^{\infty }\sin c(n\cdot DR)\cdot \sin c\cdot ((\Delta f+nf_{seq})\cdot K\cdot T_{seq})\cdot e^{j\Delta \phi }\right]$$

Finally, the accumulation-and-dump output model of the periodic pulsed navigation signal can be defined as Equation (9) by applying Equation (8) as the first term of Equation (2). Because the *DR* term in Equation (6) will affect the noise density, as well, we normalized Equation (8) by *DR* to define Equation (9).

(9) $$\begin{array}{ll} {\langle IQ\rangle }_{periodic} & =\left[\sum_{n=-\infty }^{\infty }\sin c(n\cdot DR)\cdot \sin c((\Delta f+n\cdot f_{seq})\cdot K\cdot T_{seq})\right]\\ & \cdot \sqrt{2\frac{C}{N_{0}}\cdot DR\cdot T_{seq}}\cdot R(\tau )\cdot D\cdot e^{j\Delta \phi }+\eta \end{array}$$

**Figure 4:**
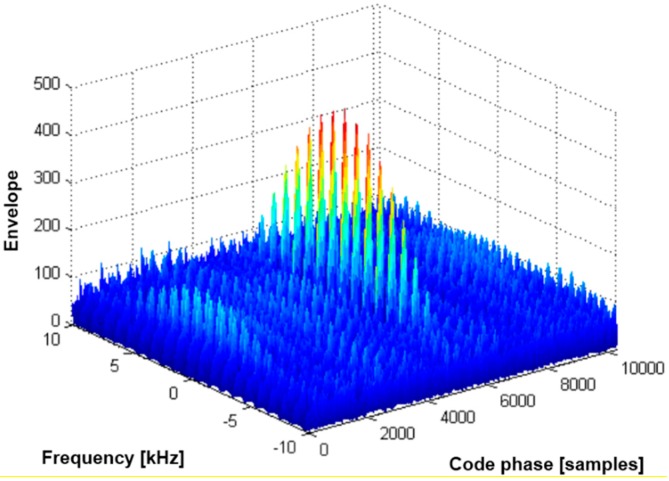
Side lobes due to periodic pulsed signals.

Equation (9) indicates that the sinc function train repeats every *f_seq_* Hz and that the overall shape on the frequency axis is limited by the sinc function presented in Equation (2). This is a type of aliasing phenomenon caused by additional periodic pulse samples, which may lead to a false lock during the signal acquisition process. In Figure 4, we showed the two-dimensional signal acquisition results as simulated using the FFT technique. A GPS C/A code-like sequence was used with a duty ratio of 0.1, and the length of the coherent integration time was two sequences. As derived by Equation (9), we note that there is a sinc function train under the wide sinc function in Figure 4. The false lock issue caused by the sinc function train (or the side lobes in the frequency domain) is a major concern in the pulsed signal processing. The various algorithms utilized in earlier work and the algorithm suggested in this paper to dissolve the false lock issue will be described in the next section.

**Figure 5:**
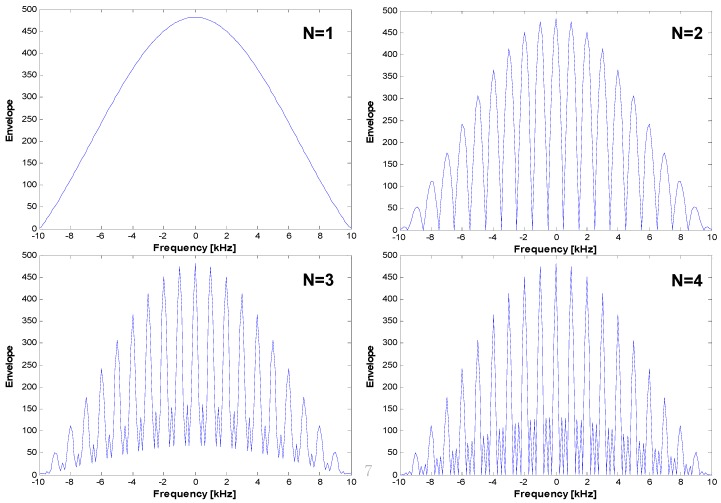
Auto-correlation function (*N* = the number of coherent integrations in sequence units).

In Figure 5, we present the signal acquisition results of pulsed signal samples with a duty ratio of 0.1 while varying the number of coherent integrations *K*. For the simulation, we used a Gold code, for which the code sequence length is 1023 chips.

The upper left figure shows the signal acquisition results when the number of coherent integrations *K* is set to one (1 ms, 1023 chips). As redefined in Equation (2), the null-to-null space is extended by the duty ratio. However, in the case of multiple coherent integrations (*K* > 1), we note that the repetition pattern of the sinc function train with *f_seq_* (*i.e*., 1 kHz) appears, as shown in both Figure 4 and Equation (9). As expected from Equation (9), the null-to-null space of the sinc train becomes narrower if *K* becomes larger. Therefore, we must use smaller frequency bins during the signal acquisition process. It is important to note that a long integration time always causes a long signal acquisition time or increased hardware complexity.

## 3. The False Lock

The pulsing scheme is known as the most effective solution to avoid interference between pseudolite signals and GNSS signals or between pseudolite signals. However, the pulsing scheme causes an additional frequency in the original signal, which leads to the false lock effect during the signal acquisition and tracking processes. As shown in Figure 5 in the previous section, the signal acquisition process in the FPGA (field programmable gate array) module may be locked onto a side lobe in the frequency domain, as the BOC (binary offset carrier) signal acquisition process experiences a false lock due to multiple side lobes in the code chip domain.

To resolve the false lock effect of the pulsed signal, previous research results, such as RTCM SC-104 or the RTCA special committee SC-159, suggested a randomized pulse sequence [11,12]. In the case of the RTCM suggestion, the pulse duration is 93 code chips, corresponding to 1/11 of a code sequence. The average duty ratio is defined as 0.1, and the pulse position is altered for every code sequence, indicating that a complete code sequence (1023 chips) can be used after a coherent integration time of 10 ms to prevent a false lock.

**Figure 6:**
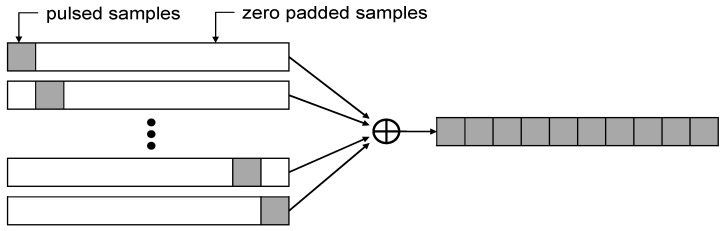
Principle of the randomized pulse sequence scheme (e.g., the RTCM SC-104 suggestion).

In Figure 6, we described how the randomized pulse sequence works to resolve the aliasing issue due to the additional frequency. In the case of the RTCM suggestion, a single pulse has 93 code chips, as explained in the above lines. The sum of 11 time slots creates a complete code sequence (1023 chips) in the correlation process, indicating that the signal acquisition and tracking process are independent of pulsing, because there are no periodic pulsed samples. The RTCA 2000 scheme uses a similar concept, but with more pseudo-randomly-distributed pulse positions. However, recently, this appears to be removed from the RTCA document due to the difficulties related to standard GPS sensors [11].

The randomized pulse position is a very useful scheme, because it can avoid the false lock effect and reduce the possibility of pulse collisions. However, a long integration time of the randomized pulse scheme (e.g., 10 or 20 ms) is not suitable for high dynamic applications, which require a limited coherent integration time. Within the framework of the ground-based radio navigation system, a deterministic pulse position scheme is assumed for signal transmissions for a similar reason. In fact, this method enables a zero pulse collision within a specific area assuming a sophisticated system design, which is another advantage for certain applications. Due to the deterministic pulse position, it was necessary to overcome the false lock issue on the navigation sensor side instead of the signal transmission side.

**Figure 7:**
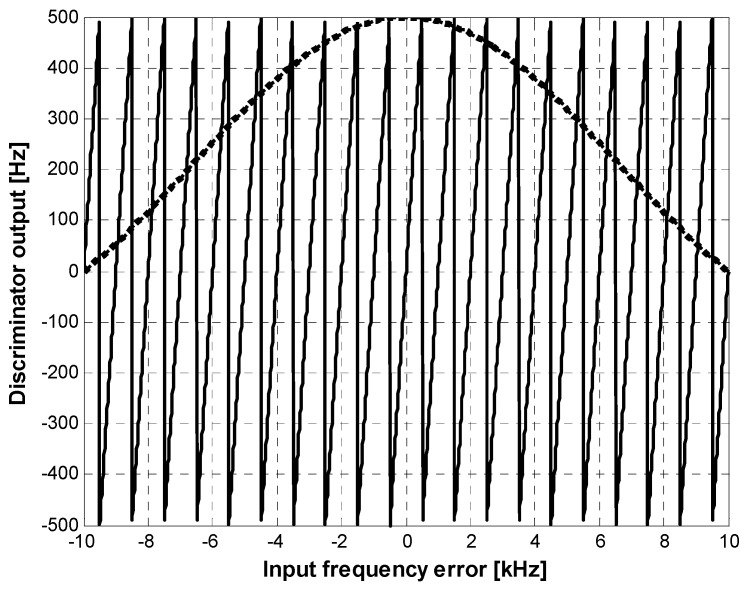
Tracking loop discriminator *vs.* correlator output.

In the tracking process, a false lock arises despite the fact that a single code sequence is used for the accumulation-and-dump process. This is clearly explained in Figure 7, which presents both the tracking loop discriminator and the accumulation-and-dump filter output in the frequency domain. In this paper, we used a second order FLL (frequency-locked loop) and the atan2 discriminator, as defined in Equation (10) [14].

(10) $$D_{FLL}=\frac{\alpha \tan2(cross,dot)}{(t_{2}-t_{1})\cdot 2\pi }$$

Here,

$$dot=I(t_{1})\cdot I(t_{2})+Q(t_{1})\cdot Q(t_{2})$$

$$cross=I(t_{1})\cdot Q(t_{2})-I(t_{2})\cdot Q(t_{1})$$

**Figure 8:**
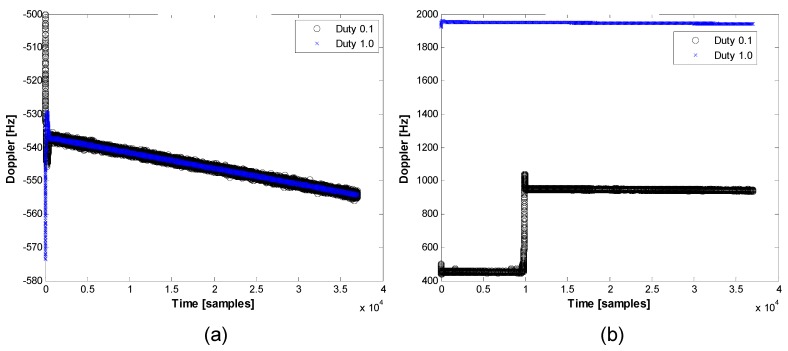
Estimated Doppler: (**a**) correctly locked; (**b**) falsely locked.

If a coherent integration time of 1 ms is used for signal tracking, from Equation (10), we can determine that the distance between lock points is 1 kHz, as presented in Figure 7. Doppler values presented in Figure 8 show what occurs when the false lock problem arises. 

Figure 8a shows that the tracking loops were locked at the correct point. The same Doppler is estimated regardless of the duty ratio value. Figure 8b shows that the tracking loop was locked in the incorrect position, in this case 1 kHz away from the true point. The false lock in the frequency domain causes two symptoms with regard to the tracking loop. First, it degrades the power (*i.e*., a relatively lower signal-to-noise ratio). Second, it gives biased velocity information to the code-tracking loop (e.g., the carrier-aided first-order DLL) and to the navigation filter.

The scale factors for the carrier-aided codes are 1/1, 540 and 1/154 for the C/A code and the P(Y) code, respectively. If there is a Doppler error of 1 kHz, the velocity biases in the code NCO are 0.65 Hz and 6.5 Hz for the C/A and the P(Y), respectively. This will likely have minor effects on the loop. However, the velocity bias is converted to approximately 190 m/s assuming the L1-band range domain. Because our system did not use the L1-band to avoid possible jamming attacks, the velocity bias will have different values with the above example; however, it is unquestionably a critical error source for the navigation filter. If the number of coherent integrations *K* is larger than one, the power degradation becomes critical, and the tracking loops may experience a frequent loss of lock. In the frequency-locked loop, the distance between the zero crossing points of the discriminator decreases; consequently, the locked point can be located in the middle of the sinc main lobe or on small side lobes.

**Figure 9:**
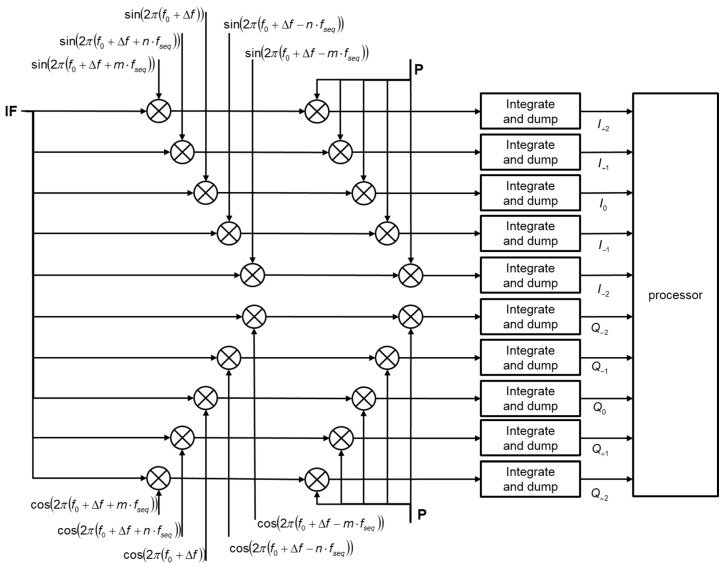
Channel block diagram for the false lock detection.

**Figure 10:**
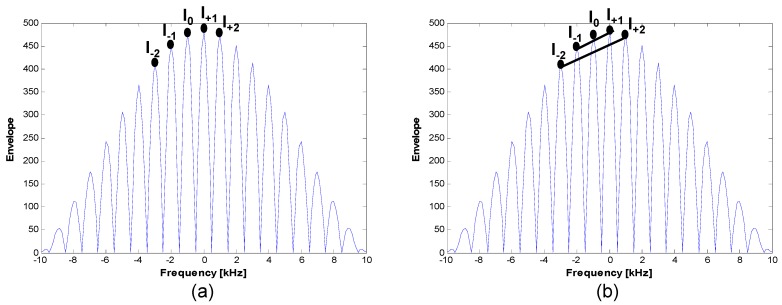
The false lock detection schemes: (**a**) The max peak finder; (**b**) The slope comparator.

To prevent a false lock in the frequency domain, we used additional correlator arms in the fast signal-acquisition module implemented in the FPGA. This concept is similar to the concept of the bump-and-jump technique, which was proposed to find a possible false lock in the code chip domain during the GNSS BOC signal acquisition and tracking processes. Figure 9 shows the proposed channel block diagram, which serves to detect a false lock in the frequency domain. The additional correlator arms located at adjacent peaks of the side frequency lobe are compared to the prompt frequency correlator arm to find the maximum peak position or they can be combined to compose more complex comparators to use the slopes between the lobes. In this paper, we refer to the former as the maximum peak finder and the latter as the slope comparator. Figure 10 presents two schemes graphically to assist with the understanding of this. The values of *n* and the *m* for frequency separation are assumed to be one for simplicity.

The maximum peak finder uses the same logic as the bump-and-jump (BJ) method suggested earlier [15], while the slope comparator is similar to the heuristic detection metrics of the SQM (signal quality monitoring) scheme with multi-correlator functionality, also proposed in an earlier work [16]. In the BJ method, early and late correlators are used in the code-tracking loop, and very late (VL) and very early (VE) correlators are used to monitor the power of other possible peaks close to the puncture correlator. Here, two additional correlator arms (VE and VL) are used only for the false lock, with a simple up/down counter mechanism applied for the decision. Counting is performed for all integrate and dump periods during the code-tracking process in the BJ. However, in the false lock detection scheme used here, the decision is designed to be made in the signal-acquisition module to reduce the computational burden. We used a hard decision rule instead of a simple up/down counting mechanism and fully utilized all correlator arms to ensure the maximum peak finding performance (e.g., five arms for I-channels, five for Q-channels). Several linear combinations to compose the heuristic test metrics have been suggested [16]. These can be categorized as follows:

(1) Delta (Δ) metrics: differential between two symmetric slopes (normalized by a puncture).
(2) Average ratio metrics: ratio between a symmetric slope and the puncture correlator.
(3) Single sided ratio metrics: ratio between a puncture and another correlator.
(4) Asymmetric ratio metrics: ratio between asymmetric correlators or ratio between an asymmetric slope and the puncture correlator.

It is challenging to find appropriate correlator positions and statistical thresholds to apply the above test metrics directly into the frequency domain. For our application, the first two metrics are appropriate, because our purpose is not to monitor signal deformation, such as an evil waveform. For high-SNR situations (e.g., during flights), the max peak finder showed generally good performance when used to detect an incorrect frequency lock.

## 4. Flight Experiment Results

**Figure 11:**
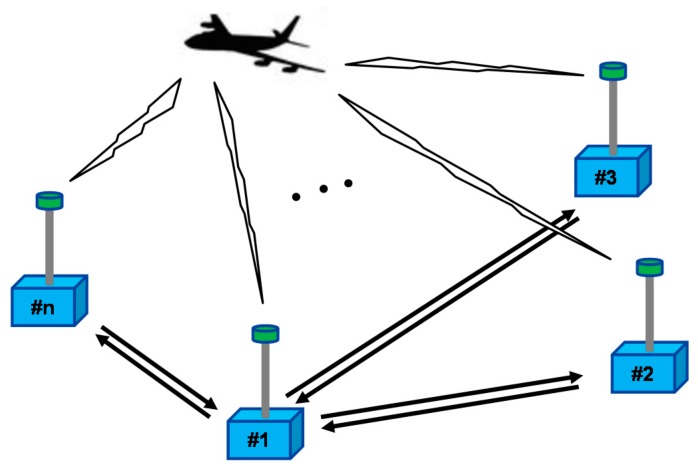
The ground-based radio navigation system using the synchronized pseudolites.

Real-time flight tests were conducted to evaluate an implemented user sensor using a ground-based radio navigation system, in this case the synchronized pseudolite navigation system shown in Figure 11. In this system, the transmission times of the pseudolites are synchronized to a specific pseudolite time known as the master pseudolite time. Detailed information about time synchronization can be found in earlier work [10].

Because the master pseudolite time is a local system time, we created a log file that included both a GPS NMEA (National Marine Electronics Association) time tag and the local system time for the purpose of analyzing the navigation accuracy performance. Assuming 10 Hz data output from a sensor, the ideal log time synchronization error from the GPS time is 0.05 s, which corresponds to one meter considering the 20 m/s dynamics of the test vehicle. We did not synchronize the local system time to the GPS time, because the basic idea of this local navigation system is to assume the vulnerability of the GNSS signal. Log synchronization using the GPS NMEA was only done for a performance analysis during the developmental stage. We did not use an IMU (inertial measurement unit) sensor to compensate for the lever arm between the local navigation system antenna and the GPS antenna. We can expect that a horizontal bias of less than one meter would be added to the navigation results due to the lever arm. In the case of the vertical direction, it can be corrected mostly with body-frame information, because the UAV helicopter is supposed to keep its pitch constant. As described earlier [10], pseudolites installed on the ground use dual-frequency bands as one of the near-far problem resolutions. During the flight test, the master pseudolite and the slave pseudolites uses different bands. Possible near-far problems between the slave pseudolite signals are assumed to be mitigated by the pulsing scheme.

To avoid cross-correlations in the satellite navigation system, pseudolites can use the frequency offset. For pseudolite sensors using both GPS and pseudolite transmitted signals, in-band offsets at satellite-signal spectral nulls are used with a single RF frontend. Because our system is an alternative navigation system, it uses out-of-band offsets to prevent hostile energy sources from interfering with the GPS signal.

**Figure 12:**
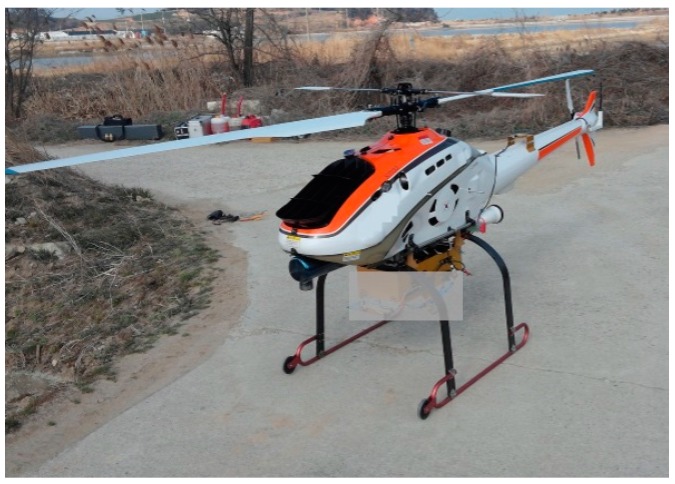
The unmanned helicopter and navigation sensor used for the flight tests.

The unmanned helicopter shown in Figure 12 was used to carry the ground-based navigation sensor during the flight test. A radio navigation sensor using a pseudolite signal from the ground does not have good vertical performance characteristics, because it has a relatively large vertical DOP (dilution of precision). Therefore, the cruise height of an air vehicle should be kept as high as possible so as to prevent poor condition numbers during the positioning calculations. During the flight test, we planned the height of the unmanned helicopter to be several hundred meters, because the safety of the vehicle should be guaranteed against strong wind. While the planned height was high enough to obtain the line of sight from all transmitters during the cruise mission, the expected vertical DOP was not good.

The pseudolites were installed in a coastal area; the horizontal distances between them were set to dozens of kilometers. The multipath signal reflected from the sea surface was one of the critical issues to resolve before the flight test could be conducted. We attempted to install ground stations as far as possible from the coast line to avoid possible direct reflections from the sea surface, and we used a spatial diversity combining technique with the time synchronization sensors installed at the same site with the transmitters, as described in previous research [10,17]. The time synchronization performance is an important measure to operate a ground-based radio navigation system within a specific accuracy level in real time. In this study, all of the ground sensors used multiple correlators in the code-tracking loop to reduce the multipath errors from inland areas. However, a simple narrow correlator was implemented in the user sensor installed in the unmanned helicopter to reduce the possibility of a loss of lock during the flight.

**Figure 13:**
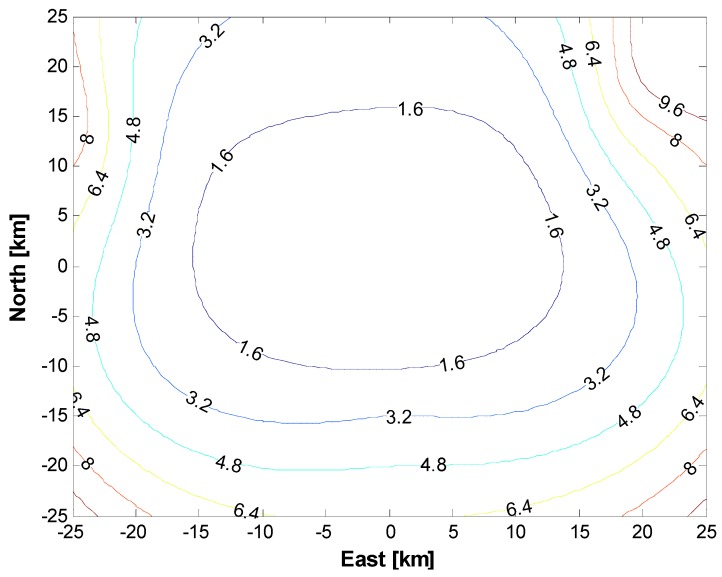
Horizontal dilution of precision (HDOP) of the test area.

Figure 13 depicts a computed version of the horizontal DOP of the flight test area. The master station is centered on four slave stations optimally placed in the test area. The DOP has a minimum value close to the master station, which is located at the center of Figure 13, and this value increases exponentially if a user vehicle moves away from the center. The trajectory of the unmanned vehicle equipped with the DUT (device under test) was scheduled to cross the center area to ascertain the condition that led to the best performance. In this paper, we assumed a horizontal DOP (HDOP) of two or less as the operational criteria to define the effective mission area of the vehicle.

**Figure 14:**
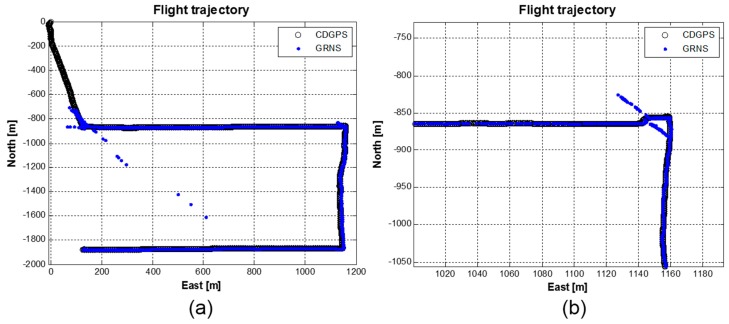
2D trajectory: (**a**) Full trajectory; (**b**) Zoom-in figure of the upper right corner of (**a**).

The real-time flight trajectories using the GRNS signals, as well as the post-processed CDGPS results using GNSS signals are shown in Figure 14 together. Position calculation in the developed sensor was done, and the results were stored at 10 Hz. In the unmanned helicopter, a NovAtel GNSS receiver was equipped to store the log files. The precise positions of the vehicle were computed to mm to cm accuracy by the GrafNav software. To provide correction information to the program, a DGPS reference station was installed very close to the master station with a NovAtel OEM6 receiver. We had conducted flight tests several times with UAV helicopters; however, from Figure 14 to Figure 16, we focused on the first flight result to explain the flight analysis clearly.

In Figure 14, we present a 2D trajectory of the first flight in ENU (east-north-up) coordinates. Figure 14a shows a full trajectory, and Figure 14b presents a zoomed-in trajectory of the right upper corner of Figure 14a to show the positioning error in detail. Very large outliers were noted, as marked with the blue dots in Figure 14a. Poor condition numbers in the least squares estimator caused the outliers, which occurred during a low-elevation stage. When the altitude of the flight vehicle is low, the navigation sensor attached to the vehicle experiences low visibility due to the geometrical environment, unstable signal power conditions and the poor DOP situation. The last factor is the most critical error source related to the outliers. The trajectory shown with the black circle only close to the upper left corner does not have a three-dimensional navigation solution due to a visibility problem (*i.e*., the low elevation). The upper right corner of Figure 14a corresponds to the best DOP area, which is above the area of the master station. At every corner of the trajectory, the unmanned helicopter stayed for some minutes in hovering mode and changed its heading to an appropriate direction. Just after the rotation of the head, the navigation sensor experienced positioning errors distributed in a specific direction, as can be seen in the corner area of the zoomed-in Figure 14b. We suspect that blockages by the leg structures of the unmanned helicopter and the gain patterns of the equipped antennas likely caused these errors during the rotation. Because we applied the spatial diversity combining technique to the stations installed on the ground and to the navigation sensor equipped on the vehicle, the performance degradation due to rotation must be minimized.

**Figure 15:**
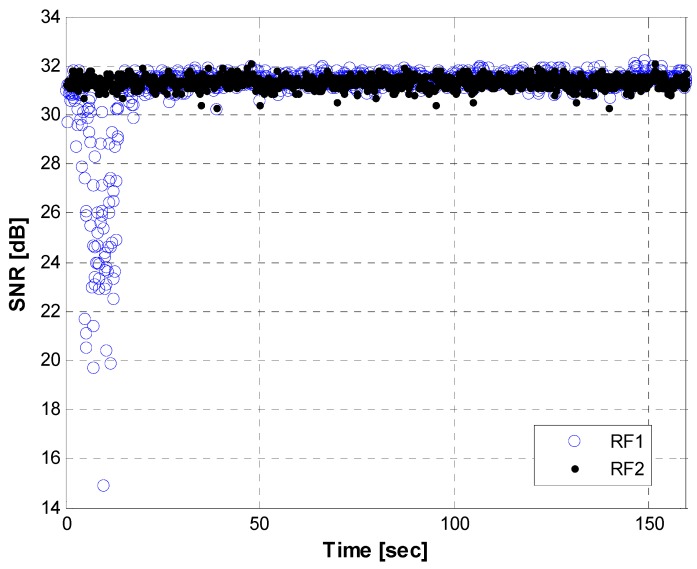
Received signal power transmitted from the master station while hovering.

**Figure 16:**
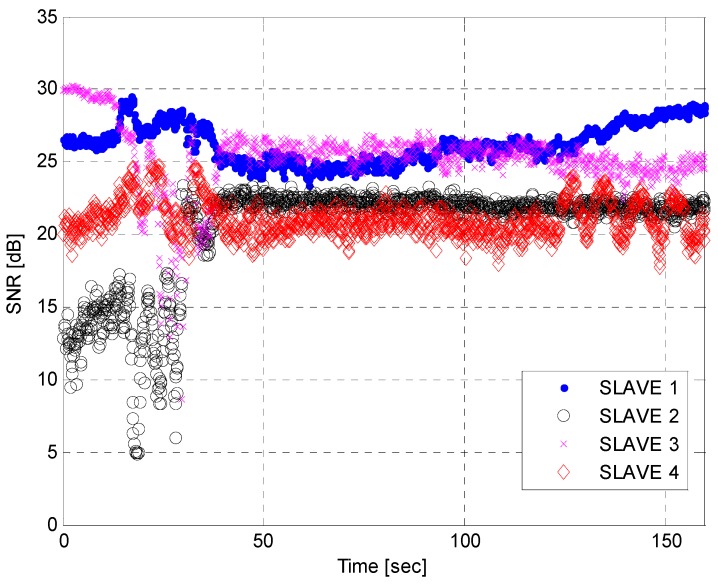
Received signal power transmitted from the slave stations while hovering.

Figure 15 and Figure 16 show SNR graphs created after the analysis of the received signal power variations during the hovering, as described in Figure 14. RF1 and RF2 in the Figure 15 denote the SNR from the first and second RF antennas used for the diversity combining process. Figure 16 presents the SNRs from a single antenna, as there was a cable connection problem at a specific RF band during the flight. As shown in the two figures, at the initial stage of hovering, the SNR values from most of the channels fluctuated severely due to the rotation of the heading. A few epochs in the RF1 of the master station and dozens of epochs from the slave Stations 2 and 3 dropped below a threshold and affect the geometry matrix for the positioning computation. Except for the rotation time (~about 40 s), the signal powers maintain relatively stable values.

**Figure 17:**
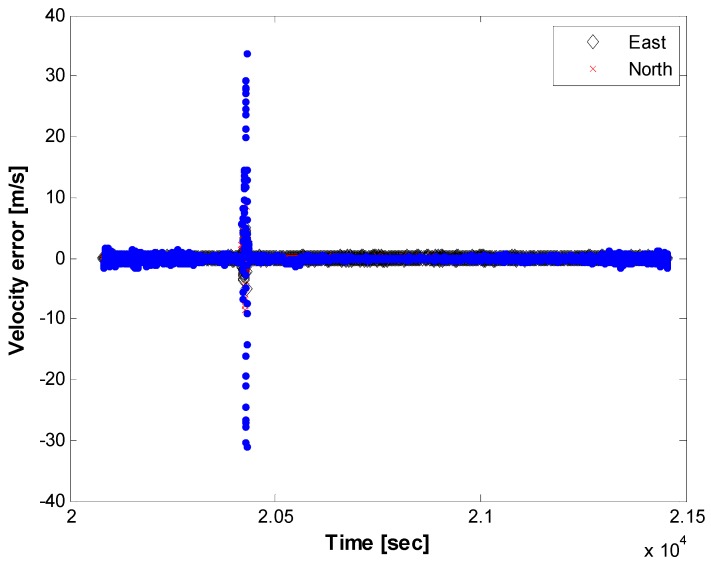
Velocity error while cruising, horizontal components only.

From Figure 14, Figure 15 and Figure 16, we can conclude that the horizontal navigation performance and the received signal power showed stable results, except at the low-elevation area with a very high DOP (e.g*.*, < several thousand PDOP) and during rotation while hovering. There were no position jumps or lock losses, which can stem from a false lock. An additional and more direct measure to check if there were false locks is to analyze the estimated velocity values, as a false lock represents the Doppler frequency bias. The velocity errors are plotted in Figure 17, for which the time scope corresponds to a cruise mode with a nearly constant altitude. Outliers caused by the rotation of the heading are visible, caused by the hovering time, as explained in Figure 15 and Figure 16. Except for the velocity outliers, the figure shows that the estimated unsmoothed velocities are stable while in cruise mode.

**Figure 18:**
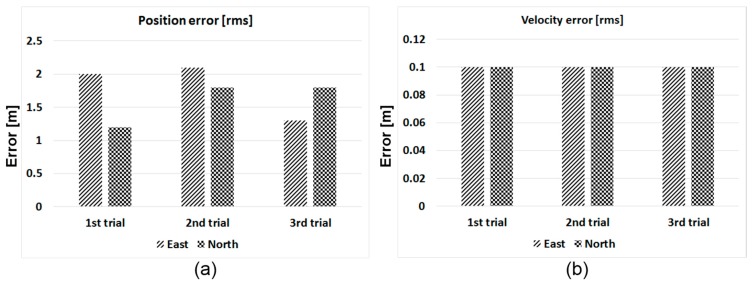
Performance analysis. (**a**) Horizontal error; (**b**) Horizontal velocity error.

During flight trials, we had conducted three sorties, and performance analysis results are plotted in Figure 18. Solutions for which horizontal DOP is less than 2.0 are included for the analysis. Due to the lever arm, each trial has a slightly different RMS error, as can be seen in Figure 18a. Mean horizontal RSS (root sum square) error was approximately 2.4 m for all flight trials. Clock synchronization error, residual tropospheric delay, tracking jitter and the lever arm can be considered as dominant error sources. In the case of velocity performance, horizontal velocity RSS error was approximately 0.14 m, and all trials show similar performances. In this paper, vertical performance was not considered for the analysis, because vertical DOP is not good in tested altitudes, as explained previously.

## 5. Conclusions

The ground-based radio navigation system is a local navigation system that uses synchronized pseudolites. It has been studied as an alternative to satellite navigation systems, such as GPS or Galileo. One of the major problems to be resolved in relation to a pseudolite-based navigation sensor is the near-far problem. The transmission of pulsed signals is known to be the most effective means of mitigating the interference caused by the near-far problem. Previous studies suggested pseudolite transmitters using a randomized pulsed sequence to overcome the aliasing issue caused by the additional frequency term of the periodic pulse. However, a randomized pulse sequence may not be adequate in some applications, due to the dwell time required to use all of the sequence chips.

This paper introduced a mathematical model for the post-correlation of the periodic pulsed signal and proposed algorithms to resolve the false lock issue due to the aliasing process. A flight test using an unmanned helicopter was conducted to verify the implemented algorithm. The results showed that there were a few outliers caused by an extremely poor DOP condition at a low elevation, as well as power fluctuations and signal blockages due to the rotation of the heading while hovering. However, the overall navigation performance was quite good, *i.e*., nearly equivalent to that of a satellite navigation system. Most importantly, there were no side effects caused by a false lock, such as a velocity bias or a frequent loss of the lock.
